# On the interchangeability of presentation order for cause and effect: Experimental tests of cue and outcome-density effects

**DOI:** 10.1177/17470218241299407

**Published:** 2024-12-09

**Authors:** Sahana Shankar, Nicola Byrom, Wijnand A P van Tilburg, Tim Rakow

**Affiliations:** 1 Psychology Department, Institute of Psychiatry, Psychology and Neuroscience (IoPPN), King’s College London, London, UK; 2 Royal Holloway, University of London, Egham, UK; 3 Department of Psychology, University of Essex, Colchester, UK

**Keywords:** Contingency learning, cause density, cue effect density, outcome density

## Abstract

Studies of cue-outcome contingency learning demonstrate outcome-density effects: participants typically overestimate contingencies when the outcome event is relatively frequent. Equivalent cue-density effects occur, although these have been examined less often. Few studies have simultaneously examined both event density effects or have manipulated the presentation order of the events, limiting knowledge of whether these phenomena share underlying principles. We report three well-powered experiments to address those gaps. Participants judged the effectiveness of a medical treatment after viewing a series of pairings for two events, a cause (treatment given vs. not) and an effect (patient recovered vs. not). Experiment 1 manipulated both event densities independently. We then manipulated the presentation order for the cause and the effect, alongside a manipulation of effect density (Experiment 2a) or cause density (Experiment 2b). Experiment 1 found a large main effect of event density ( $\eta_{p}^{2}$ = .55), which was qualified by a significant interaction between event type and density level ( $\eta_{p}^{2}$ = .10) whereby effect density had greater impact than cause density. Experiments 2a and 2b found effects for effect density ( $\eta_{p}^{2}$ = .60) and cause density ( $\eta_{p}^{2}$ = .31). The effects of cause–effect presentation order were always small and non-significant. We conclude that effect-density manipulations had substantial impact on contingency judgements, and cause-density manipulations less so. Moreover, it matters little which event (cause or effect) is seen first. These findings have implications for contingency, associative, probabilistic, and causal models of contingency judgement; primarily, that people may be more sensitive to the causal status of events than to their temporal order of presentation.

Successfully identifying associations between events is fundamental to cognition (Shanks, 2010). This form of pattern detection is a basic building block of the learning required for adaptive functioning, ranging from learning which words represent which objects or ideas, to learning which outcome(s) reliably follow a specific type of action (Batterink & Neville, 2011; Shanks, 1993; Shelton & Martin, 1992; Werker et al., 1998). Learning theorists have, therefore, sought to understand whether and how associations and causes can be accurately identified—and to identify any conditions that hinder people’s ability to accurately gauge the strength of an association or causal path between a cause and the subsequent effect. One such hindrance relates to the relative frequency, or “density,” of events. For example, when an allergic reaction occurs, individuals typically will know the effect (allergic reaction) and may also identify a putative cause (allergen). Changes to cause density (allergen prevalence) or effect density (allergy prevalence) can alter judgements of strength of association, even when the actual relationship between events remains constant.

The most prominent of such density-related biases occur when, due to high frequency of the cause or effect, a cause–effect association is perceived when none exists (Allan et al., 2005; Matute, 1995; Musca et al., 2010). This can lead people to attribute a causal relationship between two independent events, called the illusion of causality (Matute et al., 2015). This phenomenon is important because it represents a common error in human learning that is thought to contribute to the development and maintenance of superstitious beliefs and pseudoscientific thinking (Matute et al., 2015).

In this article, we examine whether biases arise in judgements of contingency as cause (cue) density and effect (outcome) density are manipulated. These manipulations are made while varying the presentation order of putative causes and effects to see how these variations influence judgements. Within much of the associative and contingency-learning literature, causes are referred to as cues while effects are referred to as outcomes. We will use the terminology of cue and outcome across this article.

Alloy and Abramson (1979) conducted one of the first studies of the outcome density-related bias, using a modified paradigm previously introduced by Jenkins and Ward (1965). In a series of contingency problem trials, participants were invited to choose whether or not to press a button to assess the relationship between button pressing and the light turning on. These researchers manipulated both the contingency between button pressing and the light turning on, as well as the overall frequency of the light turning on—referred to as the outcome density. In some conditions, the probability that the light would turn on after pressing the button was higher than for not pressing the button, that is, there was a positive contingency between button pressing and light turning on. In other conditions, the probabilities of the light turning on were equal for button presses and non-presses, equivalent to zero contingency. In some conditions, the overall probability of the light turning on was higher than 50%, whereas in others it was not, representing high and comparatively low outcome density, respectively. After completing 40 trials, participants’ judgements of the relationship between cue (button pressing) and effect (light illumination) were measured. An outcome density-related bias was found, whereby the relationship between cue and outcome was judged to be stronger when outcome density was high rather than low, even when there was no contingency between the events (Alloy & Abramson, 1979).^1^ Further studies have found outcome density-related biases to be robust across variations of the original paradigm (Blanco et al., 2013; Crump et al., 2007; Gillan et al., 2014; Matute et al., 2007; Msetfi et al., 2005; Vallée-Tourangeau et al., 2005). These variations include an allergy paradigm (assessing which foods cause allergy; Blanco et al., 2013) and a medical treatment paradigm (judging the effectiveness of a treatment for a disease; Blanco et al., 2011).

Although less frequently studied than outcome density, bias in judgement related to cue density has also been found for contingency learning. Using an allergy paradigm, Vadillo et al. (2011) manipulated cue density to be either low or high, and found a cue-density bias whereby judgements of association increased with cue density Specifically, participants believed more strongly that a substance was an allergen when that substance featured in more than 50% of learning trials, despite the contingency between substance and allergic reaction remaining constant. Perales et al. (2005) extended this work by manipulating cue density, and simultaneously varying whether or not there was a positive contingency. An effect of cue density was found, with contingency judgements being higher in the high-density conditions compared with the low-density conditions. However, while outcome density was held constant within each contingency, it varied between the positive and zero contingencies—thereby confounding contingency and outcome density and making it difficult to assess whether contingency level moderates the size of the outcome-density bias.

Few studies have simultaneously (and systematically) examined cue-density and outcome-density biases. In one such rare investigation, Blanco et al. (2013) used an allergy paradigm with zero cue-outcome contingency and employed a 2 × 2 design with cue density (high vs. low) and outcome density (high vs. low) to examine the interactive effects of cue and outcome densities. They found an effect of outcome density in all conditions, and a small effect of cue density, but only when outcome density was high. They also ran a similar experiment with a neutral scenario where the stimuli consisted of geometrical figures and found similar effects. However, Blanco et al. did not manipulate the order of cue and outcome presentation. So, while their findings may be interpreted in line with outcome- and cue-density biases, it is conceivable that these biases actually relate to the order of event occurrence; that is, manipulating the density of the “second event” to be presented may have a stronger effect than manipulating the density of the “first event” presented, regardless of whether these events are potential causes or effects. Here, an important distinction in terminology is that “cues” are not always “causes” and “effects” are not always “outcomes.” This is, because “cue” refers to the event presented first, while “outcome” refers to the event to be predicted from the cue. In most cases, this means cues are causes and outcomes are biases (i.e., following the natural order to temporal precedence). However, the order in which causes and effects are presented (i.e., made known) can be switched. If so, because the presentation order of cue (first) and outcome (last) remains unchanged, then the effect is a “cue” while the cause is an “outcome.” As such, for clarity from this point onwards, “cue” refers to the first presented event, and “outcome” is the second presented event; and rather than referring to cue and outcome-density biases, we will use the terms cause-density and effect-density biases, respectively.

## Overview of research questions and experiments

We systematically examined the relative size of cause density and effect density in contingency learning, through a three-experiment investigation that incorporated and improved upon methods used in previous studies. These experiments provided tests of contingency-learning theory, probabilistic contrast models, causal theories, and associative models. In Experiment 1, we compared cause-density and effect-density biases directly, by manipulating these event densities in a contingency-learning paradigm. By investigating cause-density and effect-density biases systematically, we investigated whether the same cognitive principles underlie both effects. If the effect sizes differ and/or one effect is conditional on the level of the other variable (with the other variable always having an effect), then this suggests that the mechanisms operating on these events are (at least partially) distinct, such as the importance of causal status assigned to causes and effects. This first experiment thus closely replicated the study by Blanco et al. (2013), who found evidence for both effect- and cause-density biases, with the former exceeding the latter in size.

After confirming the presence and relative sizes of effect and cause-density biases, we then extended our investigation in Experiments 2a and 2b by, in addition, manipulating the presentation order for the cause and the effect. Critically, this allowed us to examine the impact of presentation order independently of cause versus effect density. This extension to the contingency literature reflects that while, logically, a cause must precede an effect, sometimes people observe or learn of an effect before they see or hear of its possible causes. For example, a doctor may observe signs of infection in patients before learning about the possible causes these patients were exposed to. A manager might see that their salesforce has made a number of successful sales before they find out what steps their workers took in an attempt to achieve those successes. Some indication that the presentation order of the cause and the effect can be important for processing information comes from the causal theory literature on semantic research. Fenker et al. (2005) presented participants with pairs of word which described events as causes (e.g., rain) or effects (e.g., flood). With a temporal manipulation of these words, participants’ reactions were quicker when cause preceded effect compared with when effects preceded the cause. However, it is not clear how such an effect would translate to contingency learning; for example, whether or how the biases that are associated with increasing the event density are affected by event presentation order. Therefore, our experiments address a key aspect of theory that may have important applications beyond the laboratory.

Manipulating the event presentation order afforded the exploration of interaction effects involving both density level and event presentation order. This allowed us to test whether the influence of cause density or effect density on contingency judgement is altered by reversing the order in which event information is seen. Thus, to contrast causes and effects on the basis of their roles in defining contingencies (as distinct from their order of observation), the temporal confound must be removed. Manipulating the presentation order of causes and effects allows one to do so, and the impact of this temporal ordering can be directly assessed. For example, it may be that manipulating cause density and effect density have similar impact on contingency judgements, provided that they occupy the same temporal position (e.g., both presented last as the effect), but not when occupying different temporal positions (e.g., cause presented as cue, and effect presented as outcome). This latter ordering is the default in past studies (Blanco et al., 2013; Perales et al., 2005; Vadillo et al., 2011). We could, therefore, assess whether previous observations that outcome-density biases are larger than cue-density biases instead reflect that density biases are larger for whichever event is the cause rather than the effect. As such, we wished to explore the effect of this temporal ordering and did not know how this would affect the relative size of effects for cause and effect densities.

## General method

We examined event-density effects in three contingency-learning experiments. Manipulations of cause density and effect density were examined in the context of medical judgements about fictitious illnesses. We employed a standard contingency-learning paradigm throughout these studies with important incremental variations from one experiment to the next. We outline the common design and experiment idiosyncrasies below.

### Design

Each experiment had a 2 × 2 × 2 mixed design, with judgement of effectiveness of a medication as the dependent variable. Three factors were varied in each experiment: event manipulated, session order, and density level.

Each experiment required participants to complete two sessions, with cause density manipulated in one session and effect density manipulated in the other. Thus, the density event manipulated (e.g., cause density vs. effect density) was varied within subjects. The order of these sessions was randomly assigned and formed our second independent variable (between subjects, e.g., cause condition then effect condition vs. effect condition then cause condition). The sessions were a minimum of 3 days apart to minimise carry-over effects. Within each session, we manipulated density level to be high [0.8] versus low [0.2] for the corresponding event (see Table 1). The density levels for effect refer to the probability of effect given cause and probability of the effect given no cause (for which probabilities were identical because the cause–effect contingency was always zero). The higher the effect density the more frequently the effect occurs with and without the cause. The density levels for cause refer to the probability of the cause given the effect and probability of the cause given no effect. The higher the cause density the more frequently the cause occurs with and without the effect. Manipulating densities in this way created a low- and a high-density block of trials, each with 40 trials. When cause density was manipulated, effect density was held at a medium density of 0.5, for both high and low cause density; and vice versa when effect density was manipulated. Previous studies have often used only high and low densities (Blanco et al., 2013); for this study, we chose the non-manipulated density to be held at a medium level while the manipulated density was either high or low. We chose this set-up to reduce the possible interaction effects found previously between effect and cause densities (Blanco et al., 2013). The block shown first was chosen randomly. Thus, each participant provided a measurement for each of the four density-event by density-level combinations and did so in one of two session orders. Different medication and illness names were used for each of these four combinations of density event by density level, assigned at random (see Supplemental Table S1). The blocks were counterbalanced.

**Table 1.** Density levels across conditions. C=cause, E=effect.

Table 1a.	Low cause density at zero contingency.	
Medication	Recovery	
	Present	Absent
Present	4	4
Absent	16	16
	P(E/C) = .2	P(E~/C) = .2

*Δp* = .2 − .2 = 0.

**Table 1b.** High cause density at zero contingency.

Medication	Recovery	
	Present	Absent
Present	16	16
Absent	4	4
	P(E/C) = .8	P(E~/C) = .8

*Δp* = .8 − .8 = 0.

**Table 1c.** Low effect density at zero contingency.

Medication	Recovery		
	Present	Absent	
Present	4	16	P(E/C) = .2
Absent	4	16	P(E~/C) = .2

*Δp* = .2 − .2 = 0.

**Table 1d.** High effect density at zero contingency.

Medication	Recovery		
	Present	Absent	
Present	16	4	P(E/C) = .8
Absent	16	4	P(E~/C) = .8

*Δp* = .8 − .8 = 0.

### Participants

Participation was via Prolific.co (Prolific, 2020), an online research participant platform with approximately 100,000 registered volunteers at the time of data collection. Only those aged 18 or above and resident in the United Kingdom could participate. All participants declared they were fluent in English (Table 2 for demographics).

**Table 2.** Demographics of participants who completed both sessions for each experiment.

	Experiment 1 (*N* = 85)	Experiment 2a (*N* = 96)	Experiment 2b (*N* = 97)
Age, modal group *(range)*	21–25 *(18–70)*	26–30 *(18–71+)*	31–35 *(18–70)*
Female gender, *N (% of sample)*	50 *(58.8%)*	62 *(64.6%)*	60 *(61.9%)*

### Task and procedure

The task was based on Blanco et al. (2013; images used with permission) and was implemented via the online Gorilla experiment generator (www.gorilla.sc) (Anwyl-Irvine et al. (2018). After signing up, participants were prompted to complete the first session of the experiment. After 3 days, participants were invited to complete the second session of the experiment, which followed the same procedure as the first session. In each session, participants read an information sheet, provided consent, answered demographic questions (first session only), and then read task instructions. In the task, participants imagined that they were a doctor specialising in new illnesses and their potential treatments. The participants’ instructions explained that their patients were suffering from a (named) syndrome and may be treated with a specific medication. They then proceeded to the first trial.

**Figure 1.**
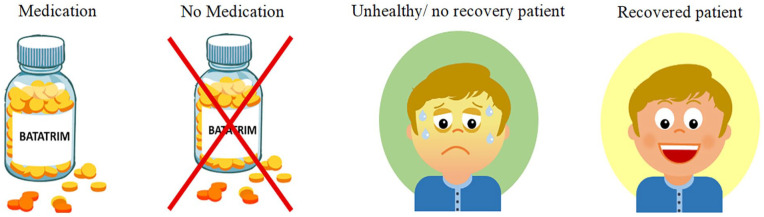
Stimuli images.

Within each trial, the following images were shown (Figure 1), in a standard presentation order: fixation cross for 750 ms, unhealthy patient image for 2,000 ms, treatment cause image (medication image vs. no medication image) for 3,000 ms, and treatment effect image (recovered patient vs. not recovered patient) for 3,000 ms. These images automatically followed each other, without input from the participant. Between trials, participants clicked “next” to continue. Each block contained 40 trials presented in a random order; each session had two blocks (low vs. high density), with block order randomised.

At the end of each block, participants judged medication effectiveness in response to the prompt: “To what extent was the medication effective in treating the illness the patients were suffering from?” To do so they used a slider, ranging from 0 (*it was not effective at all*) to 100 (*it was perfectly effective*). There was no slider handle displayed until a response was given to avoid judgement anchoring (Mussweiler et al., 2012).

Each session lasted approximately 20 min, and at the end of the entire study, participants read a short debriefing. Participants received UK £5.00 for completing the experiment. The studies received ethical approval from the Research Ethics Committee of King’s College London, MRSP-19/20-19281.

## Experiment 1: Comparing cause-density and effect-density biases

### Method

This experiment investigated the effect on contingency judgement of equivalent manipulations of cause density and effect density, to see whether, and by how much, effect-density biases and cause-density biases differ in size. Previous research found cause-density biases to be smaller than effect-density biases (Blanco et al., 2013). As such, this study adds to the literature by conducting a highly powered, within-subjects comparison of cause-density and effect-density manipulations.

#### Participants

Ninety-one volunteers participated, with 85 participants completing both sessions; see Table 2 for participant demographics. For a 2 × 2 within-subjects interaction of assuming (a) an attenuation interaction pattern, (b) any mean differences being 0.5 (all standard deviation [*SD*] = 1), (c) α = .05, (d) 40 people per group, and (e) 0.5 common correlation with no sphericity, a power of 87% is achieved. The study thus provides an adequate sample size to test the hypothesis.

#### Design and analysis

The design and procedure were as described in the “General Method” section above. Three factors were varied in the experimental design: manipulation of event (within subjects: cause density vs. effect density) by density level (within subjects: high density vs. low density) by session order (between subjects: cause-density manipulation first vs. effect-density manipulation first). Half of the sample completed the cause-density condition during the first session and the effect-density condition in the second session, with the other half of the sample completing the effect-density condition first and the cause-density second. This was included as a between-subjects factor in the analysis. By accounting for whether participants saw the cause density or event density first in the first session, the analysis automatically accounts for the condition participants were in for the second session.

We ran a 2 × 2 × 2 mixed analysis of variance (ANOVA), with the dependent variable of judgements of the effectiveness of the medication. In this factorial analysis, it is the effects involving density level that are of prime interest for our hypotheses because these relate to the impact that cause/effect density has on contingency judgements.

### Results and discussion

**Figure 2.**
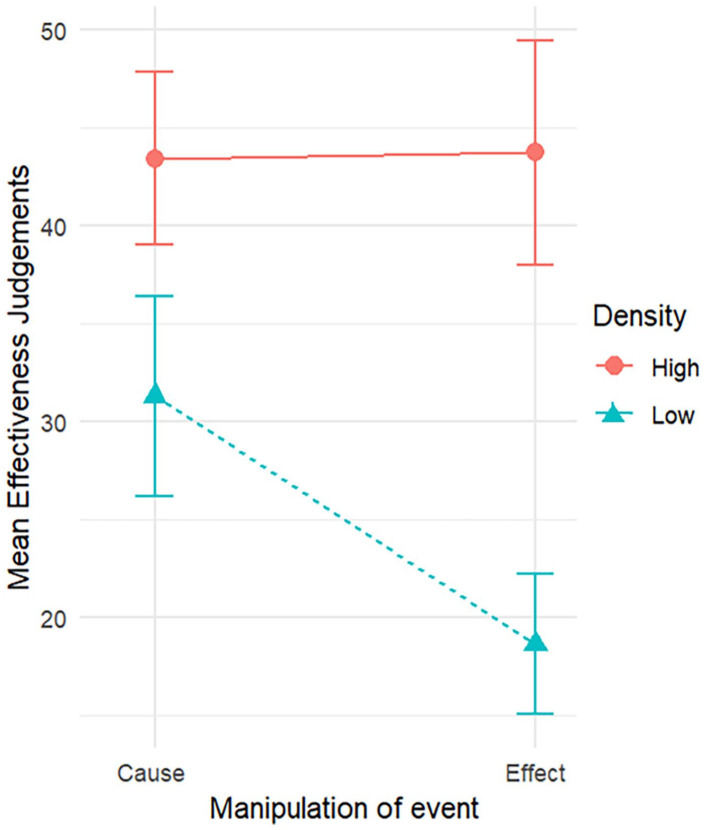
Mean effectiveness judgements by manipulation of event for high and low densities, with 95% confidence intervals.

As predicted, there was a significant main effect of density level, *F*(1, 83) = 101.91, *p* < .001, $\eta_{p}^{2}$ = .55 (very large effect). Participants judged the medication to be less effective in the low-density conditions (*M* = 24.88, *SD* = 17.22) than the high-density conditions (*M* = 43.58, *SD* = 19.06). There was a medium-sized main effect of event on the participant judgements of effectiveness, *F*(1, 83) = 11.16, *p* = .001, $\eta_{p}^{2}$ = .12. Participants judged the medication to be less effective in the effect-density condition (*M* = 31.09, *SD* = 18.06) than the cause-density condition (*M* = 37.37, *SD* = 18.38). That is, overall participants provided lower judgements of the effectiveness of the medication in conditions where the effect density was being manipulated, than conditions where the cause density was manipulated. There was an interaction effect of density level by event, *F*(1, 83) = 8.93, *p* = .004, $\eta_{p}^{2}$ = .10. As shown in Figure 2 and Table 3, the density effect, that is, the difference between judgements made in a high-density condition and judgements made in a low-density condition, was nearly three times larger in the effect-density condition compared with the cause-density condition. This comparison places the point estimate of the density effect as nearly three times larger in the effect-density condition compared with the cause-density condition.

**Table 3.** Mean (*SD*) response by group (cause then effect and effect then cause), split by session and manipulation of cause or effect.

	Judgements split by group				Overall judgements	
	Cause then effect		Effect then cause		Type of density manipulation	
	First session: Cause	Second session: Effect	First session: Effect	Second session: Cause	Cause	Effect
High-density condition	46.95 *(21.49)*	39.61 *(27.33)*	47.59 *(26.19)*	40.18 *(19.81)*	43.45 *(20.80)*	43.74 *(16.89)*
Low-density condition	27.83 *(20.34)*	17.78 *(17.19)*	19.39 *(16.69)*	34.52 *(26.72)*	31.29 *(23.95)*	18.61 *(16.85)*
95% CI for mean difference	11.69, 26.56	13.94, 29.72	19.76, 36.65	−2.50, 13.81	6.54, 17.77	19.41, 30.85
Density effect size *d*	.91	.96	1.28	.24	.54	1.49

To further understand the two-way interaction, both pairs of simple main effects were examined. Two paired-samples *t*-tests verified that the density effect was present in both the cause- and the effect-density conditions. Participants judged the effectiveness of the medication to be significantly lower in the low-density condition than the high-density condition, both in the cause-density condition, *t*(84) = 4.54, *p* *<* .001, and in the effect-density condition *t*(84) = 8.74, *p* < .001.

Paired-samples *t*-tests were also conducted to verify whether there was a significant effect of event in both the high-density and low-density condition. Participants’ judgements of effectiveness of the medication did not differ significantly between manipulations of cause and of effect in the high-density condition, *t*(84) = −.092, *p* = .927. However, judgements did differ significantly in the low-density condition, *t*(84) = 5.10, *p* *<* .001, with participants giving lower judgements for the effect-density condition than for the cause-density condition (Table 3).

**Figure 3.**
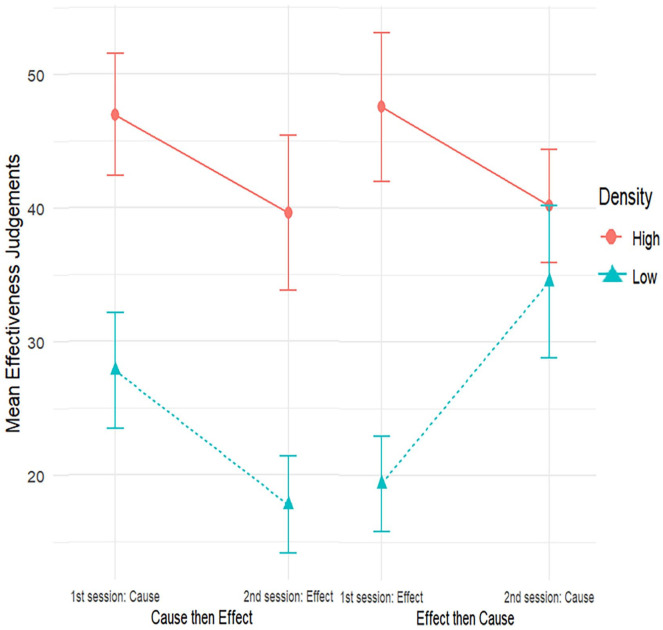
Mean effectiveness judgements by group (cause then effect, effect then cause), by session, and manipulation of event for high and low densities, with 95% confidence intervals.

We found a significant interaction between session order and density level, *F*(1, 83) = 8.93, *p* = .004, $\eta_{p}^{2}$ = .10 (medium-sized effect). This suggests that the size of the density effect varies according to the order in which events are manipulated, cause-density manipulation first or effect-density manipulation first. This two-way interaction is further qualified by the significant three-way interaction for event manipulated by density level by session order, *F*(1, 83) = 5.51, *p* = .021, $\eta_{p}^{2}$ = .06. This suggests that the differential impact on judgement of the cause-density and effect-density manipulations varied as a function of which session took place first (viz. cause condition vs. effect condition). Therefore, we compared the size of the density effect across each combination of session order and manipulation of event (Table 3 and Figure 3). This demonstrates that the density effect was present for both the cause-density and the effect-density manipulations when this manipulation took place in the first session, with both being large effects, paired-samples *t*-test for difference between high and low for cause, *t*(41) = 5.20, *p* *<* .001, and for effect, *t*(44) = 6.74, *p* *<* .001 in the first session. In the second session, the effect-density effect was present, paired-samples *t*-test for effect of density for the effect condition in the second session, *t*(41) = 5.59, *p* *<* .001, but there was no significant effect of cause-density manipulation, paired-samples *t*-test for effect of density in the cause condition in the second session, *t*(44) = 1.40, *p* *=* .169. In sum, from the two-way and three-way interactions involving density level and event presentation order, we conclude that the effect of density level is less consistent for manipulations of cause density than it is for manipulations of effect density of an equivalent scale, such that a cause-density effect on contingency judgement is only detected in some of the conditions of this experiment. Context or contrast effects can counteract the effect of cause density as indicated by the moderating effect of session order. This is in line with previous research that examined the interactive effects of cause densities and effect densities on contingency judgements, which found that cause effects where only found with high effect densities (Blanco et al., 2013).

All other main effects and two-way interactions were small and non-significant, there being no main effect of session order, *F*(1, 83) = .47, *p* = .467, $\eta_{p}^{2}$ = .006; no significant interaction between density and session order, *F*(1, 83) = .92, *p* = .342, $\eta_{p}^{2}$ = .01; and no significant interaction between event or session order, *F*(1, 83) = 1.65, *p* = .202, $\eta_{p}^{2}$ = .02 (see supplemental materials for additional analyses within each session).

In sum, we examined and compared the influence of cause density and effect density on judgements of medication effectiveness in an online contingency-learning paradigm. The findings suggest that both cause density and effect density can impact contingency judgements, but that the impact is smaller and less stable for manipulations of cause density as compared to equivalently sized manipulations of effect density.

## Experiments 2a and 2b: Density effects across event presentation orders

### Method

Experiments 2a and 2b investigated whether the impact of cause density and effect density on judgements of effectiveness of medication depends on the order in which the cause and the effect are presented. Experiment 2a investigated whether this manipulation of cause–effect presentation order moderated the effect of effect density on contingency judgement. In a symmetrical fashion, Experiment 2b investigated whether the same manipulation of cause–effect presentation order moderated the effect of cause density on contingency judgement.

A similar design to the one outlined in the “General Methods” section was employed. However, instead of manipulating the density event, the order of event presentation was manipulated. In the trials of one session, the cause was shown prior to the effect, as per Experiment 1. This thus followed the presentation of fixation cross, unhealthy patient image, treatment cause image then treatment effect image. In the trials of the other session, the effect was shown first, followed by the cause; the image order being fixation cross, unhealthy patient image, treatment effect image then treatment cause image. Participants across all conditions were given the same task instructions; there was no mention of the reverse order of events in the instructions.

### Experiment 2a: Effect density and event presentation order

#### Participants

Participants were 110 prolific members; 107 participants completed the first session, and 96 participants completed both sessions (see Table 2).

#### Results and discussion

We ran a 2 × 2 × 2 mixed ANOVA. The dependent variable was judgement of medication effectiveness. The three independent variables were event presentation order (within subjects: cause then effect vs. effect then cause), density level (within subjects: high vs. low density), and session order (between subjects: cause-then-effect in the first session vs. effect-then-cause in the first session). Effect-density biases were expected in both event presentation-order conditions.

As predicted, and replicating the findings of Experiment 1, there was a large and statistically significant main effect of effect density, *F*(1, 94) = 141.34, *p* < .001, $\eta_{p}^{2}$ = .60. Participants judged the medication to be less effective in the low-density condition (*M* = 19.16, *SD* = 11.51) than the high-density condition (*M* = 43.83, *SD* = 22.16). There was no main effect of event presentation order, *F*(1, 94) = 2.64, *p* = .107, $\eta_{p}^{2}$ = .03 (small effect); judgements of medication effectiveness did not differ significantly between the cause first (*M* = 30.13, *SD* = 16.82, 95% confidence interval [CI] = [19.77, 31.05]) and effect first (*M* = 32.86, *SD* = 16.42, 95% CI = [19.03, 28.70]) conditions. As this is a well-powered experiment with reasonably narrow CIs for means and mean differences, these findings suggest that event presentation order makes little difference to the judgement of medication effectiveness.

Our analysis showed no interaction effect of event presentation order by density level, *F*(1, 94) = 0.21, *p* = .647, $\eta_{p}^{2}$ = .002; the size of the density effect did not vary significantly by event presentation order (Table 4). Thus, neither here, nor for the main effect of event presentation order, do we find statistically significant evidence that event presentation order made a difference. In addition, there was no significant three-way interaction for event presentation order by density level by session order, *F*(1, 94) = 0.00, *p* = .972, $\eta_{p}^{2}$ = .000 (see supplemental materials for analysis on session order).

**Table 4.** Experiments 2a and 2b mean (*SD*) judgement of medication effectiveness response by event presentation order and density.

	Experiment 2a: Outcome density		Experiment 2b: Cue density	
	Cause first	Effect first	Cause first	effect first
High-density response	42.93 *(26.96)*	44.70 *(23.99)*	46.55 *(19.10)*	46.87 *(21.31)*
Low-density response	17.52 *(14.94)*	20.83 *(15.65)*	35.70 *(20.33)*	31.58 *(23.23)*
95% CI for mean difference	19.77, 31.05	19.03, 28.70	6.07, 15.61	9.63, 20.95
Density effect size *d*	1.17	1.18	.55	.69

None of the other effects in this ANOVA had any bearing on the hypotheses of this experiment, and all were small in size. There was no significant main effect of session order, *F*(1, 94) = .00, *p* = .997, $\eta_{p}^{2}$ = .000; and no significant interaction effect for density level by session order, *F*(1, 94) = .07, *p* = .791, $\eta_{p}^{2}$ = .001, or for event presentation order by session order, *F*(1, 94) = 3.29, *p* = .073, $\eta_{p}^{2}$ = .03.

Our primary interest in this experiment was whether event presentation order moderated the effect-density bias. We found no significant evidence for such moderation. However, null-hypothesis significance testing is not designed to evaluate evidence for the absence of an effect. We undertook two analyses to further evaluate how well the data aligned with the null hypothesis that the effect-density bias is not affected by the order in which events are presented.

First, we used confidence intervals to identify the plausible size of the (population) effect that event presentation order has on the effect-density bias (Greenwald, 1975). To do so, we computed the effect-density bias for each participant (i.e., the difference in judgement, for high effect-density minus low effect-density condition). The effect size for the difference in effect (cause first minus effect first) was *d* = 0.047, 95% CI = [−0.153, +0.247]. From this, we conclude that if event presentation order moderates the size of the effect-density bias, that moderating effect is likely very small (Cohen, 2013).

Second, we computed a Bayes factor to assess the evidence for the point null hypothesis of zero moderating effect of presentation order (*d* = 0) against an alternative prior (Dienes, 2014). Following the principles outlined by Dienes (2019), we used an alternative prior that was informed by our previous data for the size of the effect-density bias and by theoretical considerations that specified a reasonable range of predicted effect sizes. We, therefore, used a normally distributed alternative prior with a mean of *d* = 0 (*SD* = 0.75). As suggested by Dienes (2019), this *SD* was chosen to be half of the effect size for the effect-density manipulation that had been observed in Experiment 1.^2^ We found a Bayes factor of *BF_01_* = 6.66, demonstrating that the odds of the data are around 6 to 7 times greater under the null hypothesis than under the alternative prior that we tested. This is conventionally described as “substantial” evidence for *H_0_* over *H_1_*.

### Experiment 2b: Cause density and event presentation order

#### Participants

One-hundred volunteers participated, with 97 participants completing both sessions (see Table 2).

#### Results and discussion

We performed a 2 × 2 × 2 mixed ANOVA. The dependent variable was judgement of medication effectiveness. The three independent variables were as follows: event presentation order (within subjects: cause then effect vs. effect then cause), cause density (within subjects: high vs. low density), and session order (between subjects: cause-then-effect in the first session vs. effect-then-cause in the first session). As per Experiment 2a, it is the interaction effects involving both event presentation order and cause density that are important for the main contribution of this experiment. In line with Experiment 1, a cause-density bias was expected in both event presentation order conditions, but these biases were expected to be smaller, on average, than the effect-density bias in Experiments 1 and 2a.

Replicating the findings of Experiment 1, there was a main effect of cause density *F*(1, 95) = 42.09, *p* < .001, $\eta_{p}^{2}$ = .31 (large effect). Participants judged the medication to be less effective in the low-density condition (*M* = 33.63, *SD* = 17.34) than the high-density condition (*M* = 46.68, *SD* = 15.34). There was no main effect of event presentation order on judgements of effectiveness *F*(1, 95) = 0.89, *p* = .887, $\eta_{p}^{2}$ = .009. Participant judgement of medication effectiveness did not differ significantly between the cause first (*M* = 41.12, *SD* = 15.88, 95% CI = [37.92, 44.32]) and effect first (*M* = 39.19, *SD* = 17.02, 95% CI = [35.75, 42.62]) conditions.

There was no statistically significant event presentation order by density-level interaction, *F*(1, 95) = 1.68, *p* = .198, $\eta_{p}^{2}$ = .02 (small effect). The size of the density effect did not vary significantly by event presentation order (Table 4). Together with the absence of a main effect of event presentation order, this suggests that there is little evidence that event presentation order made a difference. There was no significant three-way interaction of event presentation order by density level by session order, *F*(1, 95) = 0.12, *p* = .731, $\eta_{p}^{2}$ = .001 (see supplemental materials for further details on session order).

None of the other effects in this ANOVA analysis have any bearing on the hypotheses of this experiment, and all were small in size. There was no significant main effect of session order, *F*(1, 95) = 2.09 *p* = .152, $\eta_{p}^{2}$ = .02; and no significant interactions for density by session order, *F*(1, 95) = 1.26, *p* = .265, $\eta_{p}^{2}$ = .01, or event presentation order by session order, *F*(1, 95) = 2.66, *p* = .106, $\eta_{p}^{2}$ = .03.

As for Experiment 2a, we re-examined the (non-significant) moderating effect of event presentation order on the cause-density effect. This moderating effect of event presentation order was small, *d* = −0.132, with a 95% CI of [−0.332, 0.068] suggesting that the moderating effect is unlikely to be greater than small-to-medium in size. Our Bayesian analysis again pitted a point null hypothesis of *d* = 0 against a normal alternative prior with *M* = 0 and *SD* of half the effect size found in Experiment 1 (*d* = 0.27). The data were again more probable under the null than under the alternative prior, with the Bayes factor of *BF_01_* = 1.35. However, because this Bayes factor is close to 1, the data are largely inconclusive for the comparison between *H_0_* and *H_1_*. Overall, the findings of Experiments 2a and 2b suggest that event presentation order makes little or no difference to the judgement of medication effectiveness or to the size of the effect-density and cause-density biases.

## General discussion

Considering all three experiments together, they replicated the previous findings for effect-density and cause-density biases in contingency learning (Blanco et al., 2013), showing that effect-density biases are larger than cause-density biases, on average. The relative difference in size appears to depend on other experimental manipulations. In addition to this, it seems event presentation order has little effect on effectiveness judgements (Table 5). Across the experiments, the size of effects for cause and effect densities was relatively consistent.

**Table 5.** Comparison of means (*SD*), CIs and effect size for cue and outcome density across Experiments 1, 2a, and 2b.

	Effect density		Cause density	
	Experiment 1	Experiment 2a	Experiment 1	Experiment 2b
High-density response	43.74 *(16.89)*	43.81 (25.46)	43.45 *(20.80)*	46.71 (20.19)
Low-density response	18.61 *(16.85)*	19.18 (15.35)	31.29 *(23.95)*	33.64 (21.87)
95% CI for mean difference	[19.41, 30.85]	[20.95, 28.32]	[6.54, 17.77]	[9.39, 16.75]
Density effect size *d*	1.49	1.17	.54	.62

The first experiment compared the effect-density bias to the cause-density bias to test whether they differed in magnitude. Consistent with the findings from Blanco et al. (2013), who conducted a similar study but with a between-subjects design with only 27 participants per condition, the effect-density bias is a robust effect. In contrast, the cause-density bias seems more dependent on context, as evidenced by the fact that the cause-density bias was present in the first session but not in the second session. The reduced cause-density bias in Session 2 could result from what was seen, or the judgement made, in Session 1. Similar cross-session context effects have been observed in other studies of judgement and preference, including when—as we did here—sessions are separated by at least 1 day (Rakow et al., 2020). We suggest that such context effects deserve further attention in contingency learning to better understand the role of relative or comparative judgements play when people assess contingency from observed or remembered samples of information (Stewart et al., 2006). Whatever the mechanism responsible, it does seem that, compared with the effect-density bias, the cause-density bias relies on a larger density manipulation to show a clear effect.

Experiments 2a and 2b examined whether the event density effect differed when the presentation order of the cause and effect was manipulated. We find it intriguing that this manipulation made no difference to the presence of density effects, and little difference to their size. For example, the finding that presentation order seems not to matter allows us to cautiously discount the possibility that the cause-density bias is smaller than the effect-density bias as a result of the cause being typically presented before the effect (i.e., designating the cause as the “cue” while the effect is the to-be-predicted “outcome”). After all, little changed when we reversed event presentation order. Moreover, the finding suggests that contingency judgements do not rely on events being encountered in their temporal order of occurrence with the putative cause preceding the effect.

Our experiments, therefore, clarify two key results that any theory of contingency learning must be able to explain. First, it clarifies that density effects in contingency learning are larger for effect-density manipulations than for cause-density manipulations. Second, it clarifies that contingency judgements and the size of density effects are mostly unaffected by the presentation order of the contingent events (cause and effect).

### Theoretical contributions

#### Contingency-learning theory

The contingency-learning account is a normative account of causal belief, stating that the belief about events should correspond with the difference in the probability that two events co-occur, *P*(Event 1|Event 2), and the probability that one of these events occurs in isolation, *P*(Event 1|No Event 2). This difference is quantified as Δ*P*—the degree of contingency (Allan, 1980). Notably, the contingency account makes no assumptions about the nature of events; all events, even causes and their consequent effects, are expected to be treated equally.

A contingency is positive if an event is more likely to result in another event, which results in a positive Δ*P*, likewise a negative contingency or negative Δ*P* occurs when the presence of an event reduces the likelihood of another event from occurring. A zero contingency occurs when Δ*P* is zero; hence, there is no difference in the probability of two events co-occurring or the one event occurring in isolation. For both positive and negative Δ*P* values, there is some relationship between the two events. Our experiments all used zero contingencies (i.e., Δ*P* = 0). As such, according to contingency-learning theory, participants should judge there to be no relationship between the two events (i.e., between the cause and effect). This was not what we observed; participants consistently identified a positive contingency between events.

There have been various attempts to use the contingency-learning account to explain density biases in judgements (e.g., Msetfi et al., 2007). These consider the influence that factors such as the inter-trial interval or response rates might have on judgements. These factors unfortunately provide us with little assistance to explain our pattern of results. We believe that the differential impact of manipulating cause density versus effect density that we observed is problematic for the contingency-learning account as a descriptive account. Put simply, it cannot explain differences in judgement between cause and effect density conditions as we have found in this article. For example, if the two events in a contingency are represented as Event 1 = cause and Event 2 = effect, there is no reason, within the contingency model, why these two events should be treated differently when calculating the probability or density of these events—they are just events. Only when contingency (ΔP) is calculated are the conditional probability of outcome given the presence of the cue, and the conditional probability of the outcome given the absence of the cue are these events treated differently. As seen in previous research (Blanco et al., 2013) when calculating event probabilities both contingency events are treated the same. To put this another way, according to the contingency table (Table 6), trials of cells B and C are identical as they are simply the occurrences of one event in the absence of the other event. Therefore, no systematic differences in contingency judgements between conditions should arise.

**Table 6.** Contingency table with cells A, B, C, and D.

Cue	Outcome	
	Present	Absent
Present	A	B
Absent	C	D

Notably, and contrary to the prescriptions of contingency-learning theory, Wasserman (1990) found that people tend to weigh the cells of a (summarised) contingency table according to A > B > C > D when judging contingencies for data presented in contingency tables. Thus, people tend to weigh cell A trials the most, while cell D trials are given the least weighting. For example, this implies in the medication treatment scenarios used in our experiments, participants will give most weight to occurrences of patients given medication and recovering, followed by patients given medication not recovering, then patients no given medication and recovery, and the least weight to patients who do not receive medication and do not recover. In addition to this, Wasserman also found that the trials of cells A and D lead to increased positive judgements while cell C and B counts decrease contingency judgements. To note, Wasserman is not examining Events 1 and 2, but rather the experiments are focusing on a cause and effect, similar to the work in our experiments. Thus, applying Wasserman’s work to our medical treatment scenario, frequent occurrences of patients given medication and recovering (i.e., cell A observations) should result in more positive judgements. Notably, increasing effect density or increasing cause density increases such occurrences. However, frequent observations of patients given medication not recovering (i.e., cell B observations) decrease judgements of contingency. Such occurrences become relatively more frequent with increasing cause density but not with increasing effect density. Therefore, Wasserman’s analysis predicts a smaller effect on contingency judgement from manipulating cause density as compared to manipulating effect density. This is because the frequent cell B observations that occur when cause density is high partially counteract the influence of the frequent cell A observations that occur when either cause density or effect density is high.

By considering the differential weighting of cells (Wasserman, 1990), the differences we found in the level of density effect between cause- and effect-density manipulations are accounted for. Specifically, when examining the low contingency conditions for both cause density (Table 1a) and effect density (Table 1c), cells A and D have the same frequency counts, while cells B and C are reversed. Therefore, considering the cell weights people apply, there are more counts of cell B for the low effect-density condition compared with the low cause-density condition, which participants would weigh more. This higher weighting would decrease the positive contingency judgements, because cells B and C decrease positive contingency judgements. This matches the findings of our experiments, whereby for the low-density conditions, participants have given more positive judgements for the low cause-density condition than the low effect-density condition.

When examining the high contingency conditions for both cause density (Table 1b) and effect density (Table 1d), again cells A and D have the same frequency counts, while cells B and C are reversed. For the high cause-density condition, there are more counts of cell B, than there are for the high effect-density condition. As such, lower judgements would be expected for the high cause-density condition, than the high effect-density condition, which also reflects the results of this study. Here, the Wasserman extension to contingency-learning theory is able to predict the findings of Experiment 1, as well as the differences between Experiments 2a and 2b for the difference in the size of the density effect.

An assumption underlying the contingency account, and other human contingency learning and association accounts, that the cause occurs first, and then, the effect occurs (De Houwer & Beckers, 2002). However, this does not fit with the data of our experiments. The data suggest that there is something fundamentally different between causes and effects, as a first event (“cue”) versus second event (“outcome”) effect was not found.

The Contingency account with the Wasserman extension may be able to account for our data. However, it must be assumed that both the cause values and effect values are encoded in memory, and the cause and effect could be observed in either order (as was the case in Experiments 2a and 2b). Therefore, if we assume the account does not need information about a cause to precede the information about effect, it can account for the findings that presentation order of cause and effect did not significantly impact the size of event-density effects.

#### Associative perspective

The associative perspective considers the associative strength of two events, the conditioned stimulus (CS/cue) and unconditioned stimulus (US/outcome). When these two events are paired, it leads to an internal connection between the events. The strength of connection is determined by how often the events are paired together and how often events occur in isolation. If context is also factored into the association between cue and US, such that we consider how context and cue might compete for associative strength, the associative account can also be influenced by how often both the cue and outcome are absent (De Houwer & Beckers, 2002). These models assume learning occurs on a conditioning trial only to the extent that the outcome is surprising. On the first conditioning trial when a cue is first paired with a US, as nothing signals the outcome, the outcome is surprising and, therefore, learning occurs. As conditioning trials proceed, cue will come to predict the outcome and the outcome will become less surprising. At some point, the cue will predict the outcome perfectly, and at this point, no further learning will occur.

Overall, the associative perspective can predict both cause-density and effect-density biases (Musca et al., 2010). However, these effects would be expected to decrease in size the more trials that occur (Musca et al., 2010; Vadillo & Barberia, 2018). This is because the overall strength of an association between a cause and an effect is determined by the predictive value of the cause, rather than the overall number of coincidences between both events. In the short term, before associative strength reaches its asymptote, the “coincidence” can have a temporary biasing effect. The effect-density bias is understood as a transient pre-asymptotic bias arising from accidental cause–effect pairings before the context becomes an effective competitor (Barberia et al., 2019). If there is unequal salience for the events of the contingency, the associative models can predict effect-density bias. Although if the occurrence of the effect is less salient than its non-occurrence, then associative models can predict positive effect-density bias. Similarly, the account can predict cause-density bias. Vadillo and Barberia (2018) demonstrated that the Rescorla–Wagner model predicts both cause-density and effect-density biases in judgement at extreme probabilities (.2 and .8) and also demonstrated differences in the sizes of these effects, with cause-density bias being much smaller than effect-density bias at both high and low probabilities. While in our studies, when the probability of cause was .2 or .8, the probability of outcome was .5, and vice versa, which does not match the exact conditions of the simulations by Vadillo and Barberia (2018), their findings reflect the differences in cause- and effect-density biases found in our data.

In regard to event presentation order, the associative account assumes that the cue is followed by the outcome, as otherwise there would be no surprise element and hence no learning. Therefore, switching the presentation order of the cue and the outcome should disrupt learning. As such, the setup of associative models would suggest that they cannot naturally account for the finding that contingency judgements were largely unaffected by reversing event presentation order to place the effect before the cause. At minimum, this suggests that for an associative account to explain our data, the cue and outcome must be arbitrarily defined—the first presented event is being associated with the second presented event regardless of the assigned labels of cause and effect. Thus, in line with our data, it would make sense to speak of surprise at cause value for a given effect value in the same way that (in the framework of the associative account) one speaks of surprise at effect value for given cause value. However, that approach to (arbitrary) definition of the cue and outcome cannot easily account for the larger size of the effect-density bias as compared to the cause-density bias. This is because, given the equal size of our cause-density and effect-density manipulations, it would not be clear why surprise-for-effect values would be larger than surprise-for-cause values. Consequently, we believe that the standard associative account does not fully account the findings of our article. However, we do note that some extended associative accounts such as the HeiDI, which considers reciprocal associations between cues and outcomes, may suggest that the order of events within a trial may not be critical for learning to take place (Honey et al., 2020), which when combined with the earlier points in our discussion suggests that the associative learning account can be adapted to the findings of this article.

#### Probabilistic contrast models

The probabilistic contrast model is another rule-based account similar to the contingency account. According to the model, individuals reason about cause–effect relationships by recalling all the events of the contingency and calculate a statistic similar to *∆P* (Cheng, 1997; Cheng & Novick, 1990). Thus, individuals compare situations that differ by whether the cause is present or absent. By comparing these situations, inferences about the relationship between cause and effect are learnt. The following equation is then used by the model,

$$P_{i}=\frac{\Delta P}{\left(1-P(e|\bar{i})\right)}$$

In this equation for the power for event i (*P_i_*), *e* is the occurrence of the effect and i is the occurrence of the event. When the cause is preventive, the denominator is $P(e|\bar{i})$ . So, the power for an event to cause an effect is a joint function of the overall contingency (*P*) and the probability of the effect in the absence of the event.

Probabilistic models can predict higher judgements of control in positive and negative contingency conditions when effect density is high. However, they cannot predict effect-density bias when the contingency is zero. However, it is argued that zero contingency is misperceived by people as being slightly positive or negative. With this assumption, the model can predict increased judgements of contingency when effect density is high (Cheng & Novick, 1990; Kutzner et al., 2008). This may, for example, occur in within-subjects designs where participants judge multiple contingencies. If this was only an issue in within-subjects designs, then effect-density bias should not be present in between-subjects designs—but it is. For our experiments, these probabilistic contrast models would predict that effect-density and cause-density biases should be small or absent in the first session. However, we found large effects. Moreover, in Experiment 1, we found a larger cause-density bias in the first session than in the second session. Although if the model was to assume that in general zero contingencies are always perceived as being either slightly negative or positive the model would provide support for the findings. Although if that is the case, there would need to be further details on when zero-contingency situations would be thought of as being negative or positive.

Similar to other models, the base assumption of probabilistic contrast models is that causes are followed by effects. Taking the probabilistic contrast model calculation as an example, power is driven by the idea that for any cause-and-effect pairing, there are always potential alternative causes. For example, if one has a fever (effect), the typically known cause may be having the flu; however, alternative causes could be an infection or heat exhaustion. Accordingly, knowledge of the base rate of the effect (i.e., the relative frequency of the effect) will let one to make a contrast between the candidate cause and alternative causes such as experimental context, and causal power is defined by contingency of an effect contrast. Therefore, this model neither explicitly states anything regarding presentation order, nor about cause-density biases. However, the findings of this article are consistent with the more general idea of the model that individuals estimate causal relationships by using information beyond the contingency information given.

#### Causal model theory

Causal model theories explain how people make statistical inferences in predictive reasoning (from observed causes to effects) and diagnostic reasoning (from observed effects to causes) (Fenker et al., 2005; Waldmann, 2000). Causal model theory proposes that individuals hold abstract knowledge about the properties of causal relations that interact with their learning of statistical information (Waldmann & Holyoak, 1992). The theory has three key assumptions. First, individuals have a predisposition to learn relationships between events as directed links from cause to effect (predictive), even in situations where information regarding the effect is presented first (diagnostic). Consequently, people make quicker and stronger predictive reasoning judgements compared with diagnostic reasoning judgements (Fenker et al., 2005). Second, the strength of the causal relationship perceived between cause and effect is dependent on the contingency between the cause and effect. The stronger the contingency between the cause and effect, the stronger that relationship is perceived to be. Finally, even though relationships between events are directional, directed from cause to effect, individuals can still make both predictive inferences and diagnostic inferences. That is, even though people are better at making inferences from causes leading to effect (predictive inference), they are still able to infer links from effects to the likely causes (diagnostic inferences).

In our experiments, causal model theory can explain positive judgement in zero-contingency conditions, due to the fact that the model assumes that people hold abstract knowledge about the world. The stimuli used in these experiments are related to medication and illness, and in general, people tend to hold the belief that medications will make one feel better and cure the illness they have. As such, the general knowledge people have about medications may have led participants to provide positive contingency judgements in the zero-contingency conditions. When considering the effect-density bias, it could be argued that participants come into the experiment assuming a positive relationship between the medication (cause) and getting better (effect) and use the information provided in the experimental conditions to update their statistical inference by each trial. This could then lead to participants giving positive but lower judgements in the low probability condition, and positive and higher judgements in the high probability judgements. Therefore, the model would be expected to predict effect-density biases. The model would also be able to predict cause-density biases, because a similar logic could be applied here. However, it is not clear to us that causal model theory would predict that cause-density biases should be smaller than effect-density biases (as was seen in our data). For example, there seems no reason to assume that a general expectation that medications are efficacious would, by itself, result in a stronger perceived causal link when effect density is high as compared to when cause density is similarly high. Therefore, if causal model theory is to explain the asymmetry in density biases, that asymmetry must presumably be a feature or bug in the process by which participants learned statistical information rather than a product of their general knowledge about medication and cure.

In regard to event presentation order, causal model theory does state that people can make inferences between causes and effects regardless of which order they are presented in. However, the model also assumes that people are predisposed to making predictive inferences from causes to effects. As such, the model implies that people should be able to make contingency judgements in both presentation-order conditions, but that the judgements might be more accurate in the predictive conditions compared with the diagnostic condition. We did find that participants made contingency judgements in both the predictive and diagnostic conditions—but with no evidence that manipulating presentation order altered their judgement. Therefore, the possibility that contingency judgements might differ between the predictive condition (cause seen first) and the diagnostic condition (effect seen first) was not supported by our findings. However, if it is instead argued that people are able to extract the information from diagnostic conditions to make the links predictive in nature, then it would be expected that the density effects would be similar across both conditions.

## Theoretical summary

Many of the learning models presented here are not able to fully account for the findings of this article. While the reasoning as to why has been outlined here, the more important consideration to take away is the fact that these models need to incorporate the assumptions that people are more sensitive to the causal status of events (i.e., whether something is a cause or effect) than to their temporal order of presentation or discovery.

### Limitations and future directions

As is common for this research on density effects in contingency learning (Blanco et al., 2013; Msetfi et al., 2007; Vallée-Tourangeau et al., 2005), we focused exclusively on zero-contingency conditions. In the interest of understanding the robustness of cause- and effect-density biases, it would—of course—be valuable to examine density effects for non-zero contingencies, both for positive and negative contingencies. Examining both direction of contingency would require adopting a bi-directional judgement scale (e.g., −100 to +100) in place of the standard 0–100 scale that we used in the current experiments, which allowed participants to consider only one direction of association. Even when negative contingencies are not examined, there are good reasons to use such a bi-directional scale. A scale bounded at zero does not permit participants to give a negative judgement, which is what the probabilistic contrast model predicts that some participants will wish to do, and which any model that allows for judgmental “noise” might expect of some participants. As such, even when there is no reason to suppose that the average participant will perceive a negative contingency when no contingency exists, providing scales that allow individual participants to make judgements in line with whatever they perceive permits better tests of theory.

Our data are in line with Wasserman’s (1990) extension to the contingency-learning model, which proposes that some cause–effect combinations receive additional (or reduced) weight when contingencies are judged. While Wasserman’s proposal predicts our data successfully, it provides a summary description (as if model) rather than a process-level explanation for our data. Thus, we do not know whether the apparent differential weighting for the A–B–C–D combinations is due to attention, memory, expectation, belief-updating, or something else. It is a challenge to understand which of these (or other) candidate processes are responsible for the density effects we have observed. However, given the large size of these effects, we think the study of density effects offers good opportunities to better understand the cognitive processes that underpin learning via experimental manipulations that affect these processes. For instance, one possibility is that participants judge contingency by trying to recall the joint distribution of cause values and effect values that they observed. Therefore, it could be valuable to test what impacts the memory for that distribution; and to see whether any biases in memory predict (or are predicted by) density effects in contingency judgement.

The session order effect that we found in Experiment 1 whereby the cause density bias was not present in the second session also points to the value of further research into the processes that underpin contingency judgement. Across all experiments, the sessions were separated by 3 days and the same density levels were used; therefore, it is not clear why this effect occurred in Experiment 1 but not Experiment 2a or 2b. One difference between Experiments 1, and 2a and 2b is that in Experiment 1 participants saw both cause and effect manipulated, while in the latter experiments, only one of these event manipulations was seen. Consequently, the session order effect seen in Experiment 1 may be due to participants seeing effect-density manipulations first, which may make participants insensitive to cause-density manipulations. To examine this further, a first step would be to establish the replicability of the order effect that we found for cause density. If found to be reliable, researchers could then examine whether—as we have suggested—judgements made in a second session are influenced by observations or judgements made in an earlier session.

The framing of the contingency rating questions in our experiments were always framed in terms of the cause leading to the effect (i.e., a “cause-framed” question, how effective was the medication in treating the illness?). Recent research suggests that effect-framed versus cause-framed questions can lead to differences in causal ratings (Camara & Livesey, 2023) with effect-framed questions leading to a smaller effect-density bias and larger cause-density bias compared with cause-framed questions. The current studies used cause-framed questions and found cause-density biases were still smaller than the effect-density biases. Therefore, the research could be expanded to examine whether using effect-framed questions reduces the size of the cause-density bias, or even eliminates it.

## Conclusion

Our research has demonstrated the robustness of effect-density manipulations on contingency judgements and their greater impact in comparison with equivalent manipulations of cause density. In addition to this, event presentation order was found to have negligible effect on judgements. Seemingly, cause-density biases in contingency judgement are reliably smaller than effect-density biases. Furthermore, contingency judgements do not seem to rely on events being encountered in their temporal order of occurrence with the cause preceding the effect. Together, these findings suggest that learning models should incorporate the assumption that people are more sensitive to the causal status of events than to the temporal order of those events.

## Supplemental Material

sj-docx-1-qjp-10.1177_17470218241299407 – Supplemental material for On the interchangeability of presentation order for cause and effect: Experimental tests of cue and outcome-density effects Supplemental material, sj-docx-1-qjp-10.1177_17470218241299407 for On the interchangeability of presentation order for cause and effect: Experimental tests of cue and outcome-density effects by Sahana Shankar, Nicola Byrom, Wijnand A P van Tilburg and Tim Rakow in Quarterly Journal of Experimental Psychology

## Notes

1. This original study shows that the tendency to ascribe cause when none existed was diminished in a sub-group of participants with depression—a finding which some describe as a demonstration of “depressive realism.”

2. With a normal alternative prior centred on *d* = 0, and because most of the density in a normal distribution lies within 2 *SD*s of its mean, this generates a reasonable range from *d* = −1.5 to *d* = +1.5 for the moderating effect of presentation order on effect-density bias. The lower bound of *d* = −1.5 represents the case that all of the effect-density bias relies on the cause being presented first rather than second. We regard that lower bound as reasonable because we know of no model that predicts a reversal of the effect density bias under any conditions. The upper bound of *d* = +1.5 reflects the case whereby the effect-density bias is substantially larger (i.e., it doubles in size) when the cause is presented after the effect as compared to when the cause is presented first as in most studies (including our Experiment 1). Between these bounds, having a normal distribution with most of its density close to zero reflects that small- and medium-sized effects are more common than large ones in psychological research.
