# Supplementary material for: On the interchangeability of presentation order for cause and effect: Experimental tests of cue and outcome-density effects
Source: Q J Exp Psychol (Hove). 2024 Dec 9;78(9):1892–908. doi: 10.1177/17470218241299407 (PMC12335625; doi:10.1177/17470218241299407)
Supplement: sj-docx-1-qjp-10.1177_17470218241299407 – Supplemental material for On the interchangeability of presentation order for cause and effect: Experimental tests of cue and outcome-density effects [file sj-docx-1-qjp-10.1177_17470218241299407.docx]

Supplementary Materials for:

On the interchangeability of presentation order for cause and effect: Experimental tests of cue and outcome density effects.

| Sahana Shankar  King’s College London & Royal Holloway University of London | Nicola Byrom  King’s College London |
| --- | --- |
| Wijnand A. P. van Tilburg  University of Essex | Tim Rakow  King’s College London |

Word count: [13100]

Sahana Shankar Psychology Department; Institute of Psychiatry, Psychology and Neuroscience (IoPPN); King’s College London; London, United Kingdom and University of Royal Holloway, Egham, United Kingdom. Tim Rakow; Psychology Department; Institute of Psychiatry, Psychology and Neuroscience (IoPPN); King’s College London; London, United Kingdom. Wijnand A. P. van Tilburg; Department of Psychology, University of Essex; Colchester, United Kingdom. Nicola Byrom; Psychology Department; Institute of Psychiatry, Psychology and Neuroscience (IoPPN); King’s College London; London, United Kingdom.

All procedures performed involving human participants were in accordance with the ethical standards of the institutional research committee. The authors declare that there are no potential conflicts of interest with respect to the research, authorship, and/or publication of this article. All authors consented to the submission of this manuscript. Corresponding author: Sahana Shankar; Psychology Department; Royal Holloway University of London, Egham; sahana.shankar@rhul.ac.uk

Instructions given to all participants across conditions of the experiments.

The instructions were also accompanied by sample images of the experimental trials images.

“Imagine that you are a doctor who works at the hospital. You specialise in new diseases and their potential treatments. The crisis induced by the new diseases may be reduced by using new treatments. However, these treatments are still in their testing phase, therefore the reliability of the treatments have not been proven yet. ### When you click the ‘Start’ button, you will be presented with a series of images which will show unhealthy patients suffering from a disease. You will also be shown information on patient recovery and any treatment the patient may have experienced. See below for images that correspond to this. After you have seen several patients, their treatments and recovery, you will be asked to assess the effectiveness of the new treatment in treating the new disease. You will be presented with 2 new diseases and their respective drug treatments. You will be given time for a break between each disease block.”

Density order effects within each session

Experiment 1

We ran a 2 × 2 × 2 mixed ANOVA with manipulation of event and density as within-subjects factors and density order during first session (low first or high first) as independent variables, and effectiveness judgments as dependent variable. There was no significant main effect of density order during first session *F*(1, 83)=2.01, *p* =.152, $\eta_{p}^{2}$ =.025. There were no interaction effect of manipulation of event × density order during first session, *F*(1, 83)=.34, *p* =.56, $\eta_{p}^{2}$ =.004, density × density order during first session, *F*(1, 83)=.55, *p* =.462, $\eta_{p}^{2}$ =.007, manipulation of event × density × density order during first session, *F*(1, 83)=.02, *p* =.887, $\eta_{p}^{2}$ =.00.

We ran a 2 × 2 × 2 mixed ANOVA with manipulation of event and density as within-subjects factors and density order during second session (low first or high first) as independent variables, and effectiveness judgments as dependent variable. There was no significant main effect of density order during second session *F*(1, 83)=1.92, *p* =.170, $\boldsymbol{\eta}_{\boldsymbol{p}}^{\boldsymbol{2}}$ =.02. There was a significant interaction effect of manipulation of event × density order during second session, *F*(1, 83)=5.63, *p* =.020, $\boldsymbol{\eta}_{\boldsymbol{p}}^{\boldsymbol{2}}$ =.06. There was no significant interaction effect of density × density order during second session, *F*(1, 83)=3.72, *p* =.057, $\boldsymbol{\eta}_{\boldsymbol{p}}^{\boldsymbol{2}}$ =.04, manipulation of event × density × density order during second session, *F*(1, 83)=1.38, *p* =.243, $\boldsymbol{\eta}_{\boldsymbol{p}}^{\boldsymbol{2}}$ =.02.

Experiment 2a: Effect density

We ran a 2 × 2 × 2 mixed ANOVA with event presentation order and density as within-subjects factors and density order during first session (low first or high first) as independent variables, and effectiveness judgments as dependent variable, to investigate whether density order during first session has an order effect. There was a significant main effect of first session density order, *F*(1, 94)=4.22, *p* =.043, $\eta_{p}^{2}$ =.043, participants gave lower judgments if they were in the low density first condition (M=28.35, SD=20.60), compared to the high density first condition (M=34.27, SD=19.34), suggesting there may have been an anchoring effect present that is based on the first judgment they made. There was a significant 3 way interaction of event presentation order × density × density order during first session, *F(*1, 94)=.9.30, *p* =.003, $\eta_{p}^{2}$ =.09 and presentation order × density order during first session, *F(*1, 94)=12.05, *p* <.001, $\eta_{p}^{2}$ =.11. There was no interaction effect of density × density order during first session, *F(*1, 94)=1.14, *p* =.290, $\eta_{p}^{2}$ =.01.

We also ran a 2 × 2 × 2 mixed ANOVA with event presentation order and density as within- subjects factors and density order during second session (low first or high first) as independent variables, and effectiveness judgments as dependent variable. There was no significant main effect of density order during second session *F*(1, 94)=1.18, *p* =.280, $\eta_{p}^{2}$ =.01. There were no significant interaction effects of event presentation order × density order during second session, *F*(1, 94)=.80, *p* =.374, $\eta_{p}^{2}$ =.01, or density × density order during second session, *F*(1, 94)=.043, *p* =.836, $\eta_{p}^{2}$ =.00, manipulation of order of event × density × density order during second session, *F*(1, 94)=.07, *p* =.789, $\eta_{p}^{2}$ =.00.

Experiment 2b: Cause density

We ran a 2 × 2 × 2 mixed ANOVA with event presentation order and density as within-subjects factors and density order during first session (low first or high first) as independent variables, and effectiveness judgments as dependent variable, to investigate whether density order during first session has an order effect. There was no significant main effect of first session density order, *F*(1, 95)=0.19, *p* =.665, $\eta_{p}^{2}$ =.002,judgmentjudgment. There was a significant 3 way interaction of event presentation order × density × density order during first session, *F(*1, 95)=6.13, *p* =.015, $\eta_{p}^{2}$ =.06. There was no interaction effect of density × density order during first session, *F(*1, 95)=1.98, *p* =.163, $\eta_{p}^{2}$ =.02. , or no interaction effect of presentation order × density order during first session *F(*1, 95)=2.82, *p* =.097, $\eta_{p}^{2}$ =.03.

We also ran a 2 × 2 × 2 mixed ANOVA with event presentation order and density as within- subjects factors and density order during second session (low first or high first) as independent variables, and effectiveness judgments as dependent variable. There was no significant main effect of density order during second session *F*(1, 95)=.14, *p* =.712, $\eta_{p}^{2}$ =.00. There were no significant interaction effects of event presentation order × density order during second session, *F*(1, 95)=.114, *p* =.736, $\eta_{p}^{2}$ =.00, or density × density order during second session, *F*(1, 95)=.010, *p* =.923, $\eta_{p}^{2}$ =.00, manipulation of order of event × density × density order during second session, *F*(1, 95)=.028, *p* =.895, $\eta_{p}^{2}$ =.00.
